# Type I Interferons induce endothelial destabilization in Systemic Lupus Erythematosus in a Tie2-dependent manner

**DOI:** 10.3389/fimmu.2023.1277267

**Published:** 2023-12-14

**Authors:** Carlos Rafael-Vidal, Sara Martínez-Ramos, Beatriz Malvar-Fernández, Irene Altabás-González, Coral Mouriño, Douglas J. Veale, Achilleas Floudas, Ursula Fearon, José María Pego Reigosa, Samuel García

**Affiliations:** ^1^ Rheumatology and Immune-mediated Diseases Group, Galicia Sur Health Research Institute (IIS Galicia Sur), Vigo, Spain; ^2^ Rheumatology Department, University Hospital of Vigo, Vigo, Spain; ^3^ Molecular Rheumatology, Clinical Medicine, Trinity Biomedical Science Institute, Dublin, Ireland; ^4^ European Alliance of Associations for Rheumatology (EULAR) Centre for Arthritis and Rheumatic Diseases, St Vincent’s University Hospital, University College Dublin, Dublin, Ireland; ^5^ School of Biotechnology, Dublin City University, Dublin, Ireland

**Keywords:** type I interferons, systemic lupus erythematosus, Tie2, endothelial destabilization, angiopoietins, cardiovascular risk

## Abstract

Endothelial cell (EC) dysfunction is a hallmark of Systemic Lupus Erythematosus (SLE) and Tie2 is a receptor essential for vascular stability. Inflammatory processes promote inhibition of Tie2 homeostatic activation, driving vascular dysfunction. In this work we determined whether type I Interferons (IFN) induce Tie2 signalling-mediated endothelial dysfunction in patients with SLE. Serum levels of Angiopoietin (Ang)-1, Ang-2 and soluble (s)Tie1 in patients with SLE and healthy controls were measured by ELISA. Monocytes from patients with SLE and Human Umbilical Vein EC (HUVEC) were stimulated with IFN-α, IFN-β (1000 I.U.) or SLE serum (20%). mRNA and protein expression, phosphorylation and translocation were determined by quantitative PCR, ELISA, Western Blot, flow cytometry and confocal microscopy. Viability and angiogenic capacity were determined by calcein and tube formation assays. We found that sTie1 and Ang-2 serum levels were increased and Ang-1 decreased in patients with SLE and were associated with clinical characteristics. Type I IFN significantly decreased Ang-1 and increased Ang-2 in monocytes from patients with SLE. Type I IFN increased sTie1 and Ang-2 secretion and reduced Tie2 activation in HUVEC. Functionally, type I IFN significantly reduced EC viability and impaired angiogenesis in a Tie2 signalling-dependent manner. Finally, SLE serum increased Ang-2 and sTie1 secretion and significantly decreased tube formation. Importantly, Tie1 and IFNAR1 knockdown reversed these effects in tube formation. Overall, type I IFN play an important role in the stability of EC by inhibiting Tie2 signalling, suggesting that these processes may be implicated in the cardiovascular events observed in patients with SLE.

## 1 Introduction

Systemic lupus erythematosus (SLE) is a complex and heterogeneous autoimmune disease characterized by the production of autoantibodies, leading to systemic and multi-organ involvement (1). SLE mainly affects women with a proportion 9:1 with respect to males and the onset is commonly the third decade of age. Despite the advances achieved in the last years for the treatment of SLE, patients still have a mortality rate 2-5 times higher than average (2). Numerous factors such as genetic, epigenetic and environmental are involved in the pathogenesis of SLE. One of the hallmarks of SLE is the imbalance between apoptotic debris and rate of its clearance, which leads to self-antigen availability and the loss of tolerance in T and B cells and to the production of autoantibodies. These autoantibodies form immune complexes, which are deposited in the tissues causing inflammation and potential damage (3).

Cardiovascular events (CVE) are one of the main comorbidities observed in SLE and are one of the leading causes of death among these patients; in fact, recent meta-analysis have shown that patients with SLE have approximately a 2.5 times increased risk of CVE in comparison to the general population (4). Traditional risk factors, including smoking, diabetes mellitus, age and hypertension are involved in the CVE observed in patients with SLE (5), but they do not explain completely the higher incidence of CVE in that patients (6). Therefore, there are other factors that contribute to the endothelial dysfunction and destabilization, which lead to the apparition of CVE (7). Recent studies have identified several of these factors involved in SLE endothelial destabilization, such as system activation, inflammation, metabolic dysregulation and decrease in endothelial progenitor cells (EPC) (7, 8).

Type I interferons (IFN) are a family of cytokines with a key role in the pathogenesis of SLE. Type I IFN signal through the IFN α/β receptor 1 and receptor 2 (IFNAR1-IFNAR2) and play different roles in SLE, including the activation and skewing of myeloid cells to antigen presenting cells, which lead to the hyperactivation of T and B cells and the production of antibodies (9). The main type I IFN producers in SLE are neutrophils and macrophages, being able to induce the IFN gene signature in endothelial cells (EC), contrary to the idea of the last decades that main producers were the plasmacytoid dendritic cells (pDC) (9, 10). Importantly, type I IFN are involved in the higher risk of CVE observed in patients with SLE (11). These cytokines induce vascular and endothelial destabilization through several pathways, such as triggering the release of inflammatory mediators and enhancing apoptosis of endothelial cells (11, 12). On the other hand, type I IFN decrease the number and reduce the functional capacity of endothelial progenitor cells in SLE, which are a key cell population involved in endothelial stability (12).

Tie1 and Tie2 are two membrane tyrosine kinase receptors, predominantly expressed by endothelial cells, with a prominent role in vascular development and homeostasis (13). Tie2 binds to different ligands, mainly angiopoietin 1 (Ang-1) and angiopoietin 2 (Ang-2), while Tie1 was consider as an orphan receptor. Nevertheless, a recent study has identified Leukocyte cell-derived chemotaxin-2 (LECT2) as a ligand for Tie1 (14). The Tie2/angiopoietin signalling is essential for vascular stability. Under homeostatic conditions, both Ang-1 and Ang-2 activate Tie2 signalling and induce vascular stabilization in a Tie1-dependent manner. Briefly, Ang-1 and Ang-2 phosphorylate Tie2, leading to the PI3K/PKB-dependent phosphorylation of the transcription factor Forkhead box protein O1 (FOXO1), which trigger its nuclear exclusion and degradation, increasing the expression of genes that promote vascular stabilization. However, under inflammatory conditions, such as those mediated by LPS or TNF, Tie1 is cleaved, triggering the inhibition of the Tie2 signalling and finally leading to vascular destabilization and dysfunction. Importantly, in this inflammatory environment, Ang-2 acts as an antagonist of Tie2, and there is a release of Weibel-Palade bodies containing Ang-2, which promotes a positive feedback loop effect (15, 16).

Different studies have shown that Tie2/angiopoietin signalling is dysregulated in SLE. Ang-1 levels are decreased and Ang-2 levels are increased in serum of patients with SLE compared to healthy controls (HC) (17). In addition, Ang-2 levels positively correlate with disease activity parameters such as the SLE Disease Activity Index (SLEDAI) (17, 18). Remarkably, Tie2 dysregulation has been implicated in both atherosclerosis and thrombosis, as well as in functional processes such as migration and angiogenesis, demonstrating that Tie2 has a prominent role in cardiovascular events that are dysregulated in patients with SLE (19, 20).

Due to critical role of inflammation on Tie2-mediated vascular impairment, in this study we determined the involvement of Tie2 signalling in the type I IFN-induced vascular destabilization observed in SLE pathology.

## 2 Methods

### 2.1 Patients and collection of samples

Patients included in this study fulfilled the American College of Rheumatology (ACR)/European Alliance of Associations for Rheumatology (EULAR) criteria for SLE (21) and supplied written informed consent, which was approved by the Ethics Committee of Galicia (study number 2020/158).

Serological analyses were performed to all patients, where we measured the level of SLE activity of every patient at the time of the visit when the sample was collected. We used the SLEDAI (SLE Disease Activity Index) and the SLEDAS (SLE Disease Activity Score) activity scores, as well as the states of low disease activity and remission by using the LLDAS and the 2021 DORIS-21 remission definitions, respectively (22–24). Also, we measured C3, C4, and anti-dsDNA antibodies as serological markers of SLE activity and we collected information about antiphospholipid antibodies (Ig M and Ig G anticardiolipin antibodies, Ig M and Ig G anti-β2 glycoprotein 1 antibodies and lupus anticoagulant) at the time of the visit.

### 2.2 Monocytes and macrophages stimulation

Peripheral blood mononuclear cells (PBMCs) were obtained from blood of healthy controls (HC) and patients with SLE using Lymphoprep (StemCell Technologies). Monocytes were isolated with MagniSort™ Human Pan-Monocyte Enrichment Kit (ThermoFisher) and cultured in Iscove’s Modified Dulbecco’s Medium (IMDM, Lonza) supplemented with 10% Fetal Bovine Serum (FBS, Corning™), 1000 I.E. penicillin/streptomycin (Lonza). Monocytes were stimulated with IFN-α (1000 I.U., PBL Assay Science) or IFN-β (1000 I.U., R&D systems) for 4 and 24 h. Alternatively, monocytes were differentiated into macrophages in the presence of M-CSF (25 ng/mL, PreproTech) for 7 days prior to stimulation with Ang-1 (200ng/mL, R&D systems) or Ang-2 (200 ng/mL, R&D systems) for 4 and 24 h.

### 2.3 Endothelial cells and stimulation

Human Umbilical Vascular Endothelial Cells (HUVEC) (Lonza) were cultured to confluence in EGM™ Endothelial Cell Growth Medium BulletKit™ (Lonza) and media was refreshed every 3 days. Cells were cultured in a humidified incubator at 37 °C and 5% CO_2_ and passages from 2 to 12 were used. Stimulations were performed at different time points with IFN-α (1000 I.U.), IFN-β (1000 I.U.), SLE serum (20% v/v) or the supernatants (25% v/v) from non-stimulated (conditioned media -CM_Med_) or IFN-β (1000 I.U.) stimulated (CM_IFN-β_) SLE monocytes.

### 2.4 HUVEC siRNA transfection

HUVEC were transfected using Dharmafect (Dharmacon) and OPTI-MEM (ThermoFisher Scientific) following the manufacturer’s protocol. HUVEC were transfected with Dharmafect for 6 hours in OPTI-MEM serum-reduced medium with Tie1 and IFNAR1 and control non-targeting (Sc) siRNAs (all 100 nM, Dharmacon). OPTI-MEM media was changed to EGM™ media and experiments were performed 48–72 h after transfection. Efficiency of transfections was tested by qPCR and western blot and is shown in **Supplementary Figure S1.**

### 2.5 RT-PCR and qPCR

RNA from monocytes, macrophages and HUVEC was isolated using the Nucleospin RNA/Protein mini kit (Macherey-Nagel). Reverse transcription of total RNA was performed using iScript (Bio-Rad) and PCR assays were performed in duplicates using SYBR green (Bio-Rad) and specific primers (Integrated DNA Technologies; **Supplementary Table S2**) with a CFX96 Touch-Real-Time PCR system (Bio-Rad). Relative gene expression was normalized to *B2M* and *GAPDH* housekeeping genes expression. The relative expression and quantity (RQ) of mRNA were calculated using the formulas 2^–ΔCtx 1000^ or 2^–ΔΔCt^, respectively. IFN signature was evaluated in PBMCs from HC and patients with SLE using expression data of *IFI44L*, *ISG15*, *IFIT2*, *IFIT3* and *MX1* and obtaining a *Z score* to classify the patients (25).

### 2.6 Enzyme-linked immuno-sorbent assay

Soluble Tie1 (sTie1), Ang-1 and Ang-2 levels in serum and cell supernatants were measured by ELISA using commercial kits as per the manufacturer’s instructions (R&D systems).

### 2.7 Immunoblotting

HUVEC were lysed in Laemmli buffer and equal amount of total protein was subjected to electrophoresis of 10% acrylamide/Bis (Bio-Rad) gels and proteins were transferred to PVDF membranes (Millipore). Membranes were incubated overnight at 4 °C with primary antibodies specific to Tie1, Tie2, IFNAR1 and Tubulin (all from R&D systems). Membranes were then washed and incubated in Tris buffered saline–Tween containing a horseradish peroxidase–conjugated secondary antibody. Protein was detected with peroxide solution and luminol enhancer solution (ThermoFisher Scientific) using a Chemi-Doc MP imaging system (BioRad). Densitometry analysis was performed with Image-J software (26). Relative protein expression was normalized to the total expression of tubulin.

### 2.8 FOXO1 translocation

3x10^4^ HUVEC were seeded in Nunc Lab-Tek 8-well plates (Thermofisher) and were stimulated with IFN-β for 15 min. Then, cells were washed with PBS, permeabilized with PBS 0.1% Triton-X for 5 min and blocked with PBS 1% BSA for 1 h. Cells were incubated at 4 °C for 1h with primary antibody specific to FOXO1 (1:100, Cell Signalling), washed and incubated with an Alexa Fluor 568 conjugated secondary antibody for 1 h in the dark. Next, an incubation for 5 min with DAPI was performed and samples were stored in PBS at 4 °C. Images were taken using a Confocal Microscope Stellaris 8 (LEICA). Images were processed and analysed with Image-J software (26).

### 2.9 Two-Photon Fluorescent lifetime imaging microscopy

Two-Photon FLIM (2P-FLIM) was used to measure the metabolic profile of HUVEC stimulated with IFN-β (1000 I.U.) for 5 h. This technique is able to perform this metabolic assessment on a single cell level without the need for labeling with external fluorescent probes (27, 28). 2P-FLIM is able to capture the fluorescence levels of protein-bound NAD(P)H, that are employed in oxidative phosphorylation, versus levels of free NAD(P)H, which are not being employed in energy production. Thus, a decrease in the signal lifetime indicates higher levels of free NAD(P)H and, therefore, lower energy production and lower metabolic activity of cells. Average lifetime (τ_avg_) of NAD(P)H for each pixel was calculated by a weighted average of both free and bound lifetime contributions. 2P-FLIM assays were performed in a custom upright (Olympus BX61WI) laser multiphoton microscopy system equipped with Titanium:sapphire laser (Chameleon Ultra, Coherent^®^), water-immersion 25x objective (Olympus, 1.05NA) and temperature controlled stage. To carry out the experiment, two-photon excitation of NAD(P)H and GFP fluorescence was performed at excitation wavelengths of 760 nm and 920 nm, respectively. Next, 2P-FLIM protocol was performed as described (29).

### 2.10 Viability assay

HUVEC were seeded in 96-well plates at 1 x 10^4^ cells/well and were stimulated with IFN-α (1000 I.U.) and IFN-β (1000 I.U.) for 24, 48 and 72 h. Next, cells were incubated with Calcein-AM (1 μM, Invitrogen) for 2 h, followed by fluorescence measurement (excitation range 490 nm, emission range 520 nm).

### 2.11< Tube formation assay

HUVEC tube formation assays were performed as previously described. In brief, 50 μL of Matrigel (ThermoFisher) were added to cold 96-well plates and left to polymerise in an incubator for 30 min at 37 °C, 5% CO_2_. 3 x 10^4^ HUVEC were seeded per well and immediately treated with IFN-β (1000 I.U.), SLE serum (20% v/v) or the supernatants (25% v/v) from non-stimulated (conditioned media -CM_Med_) or IFN-β (1000 I.U.) stimulated (CM_IFN-β_) SLE monocytes for 24h.

Alternatively, HUVEC were pre-treated with IFN-β (1000 I.U.) for 24 h and stained with CellTrace™ Far Red (ThermoFisher), while non-pre-treated HUVEC were stained with CFSE CellTrace™ (ThermoFisher) as per the manufacturer’s instructions. Images were taken using a phase-contrast microscope or an Olympus IX81-long focal length Fluorescent Microscope. Images were processed with Image-J software (26) and quantification of tube nodes was performed with the Angiogenesis Analyzer for Image-J (30).

### 2.12 Flow cytometry

HUVEC were seeded at 3 x 10^4^ cells/well in Matrigel-precoated 96-well plates to perform tube formation assay and were stimulated with IFN- β (1000 I.U.) for 5 h and 24 h. Accutase enzyme (ThermoFisher) was used to transfer cells from angiogenesis to flow cytometry plates. Live/Dead fixable NIR (ThermoFisher) viability dye was used and Fc‐γ receptor (FcγR) blocking was performed with human FcγR‐binding inhibitor (TruStain FcX Receptor blocking solution, BioLegend) prior to antibody staining. The following antibodies were used: ICAM, CD141, Claudin5, VCAM (BioLegend). Samples were acquired using a Cytek Aurora Flow Cytometer. Cell populations were analysed with Flowjo (V.10) software, using fluorescent minus one (FMO) gating controls. Results were expressed as Median Intensity Fluorescence (MFI) or the percentage of positive cells.

### 2.13 Gene expression from profiling data

Expression of Tie2 and angiogenesis related genes were retrieved from sequencing and array profiling data available on the Gene Expression Omnibus (GEO–NCBI) (GSE173767, GSE86423, GSE157293 and GSE112943).

### 2.14 Statistical analysis

Statistical analysis was performed using GraphPad Prism version 8. Normality was analysed by Kolmogorov-Smirnov test. Potential differences in patient groups and HUVEC conditions were analysed using non-parametric unpaired T test, Mann-Whitney test, Kruskal-Wallis test, parametric Paired T test, One-way and Two-way ANOVA and r Pearson correlation, as appropriate. P values less than 0.05 were considered statistically significant.

## 3 Results

### 3.1 Tie2 signalling is destabilized in patients with SLE and is associated with interferon signature and clinical characteristics

**Figure 1:**
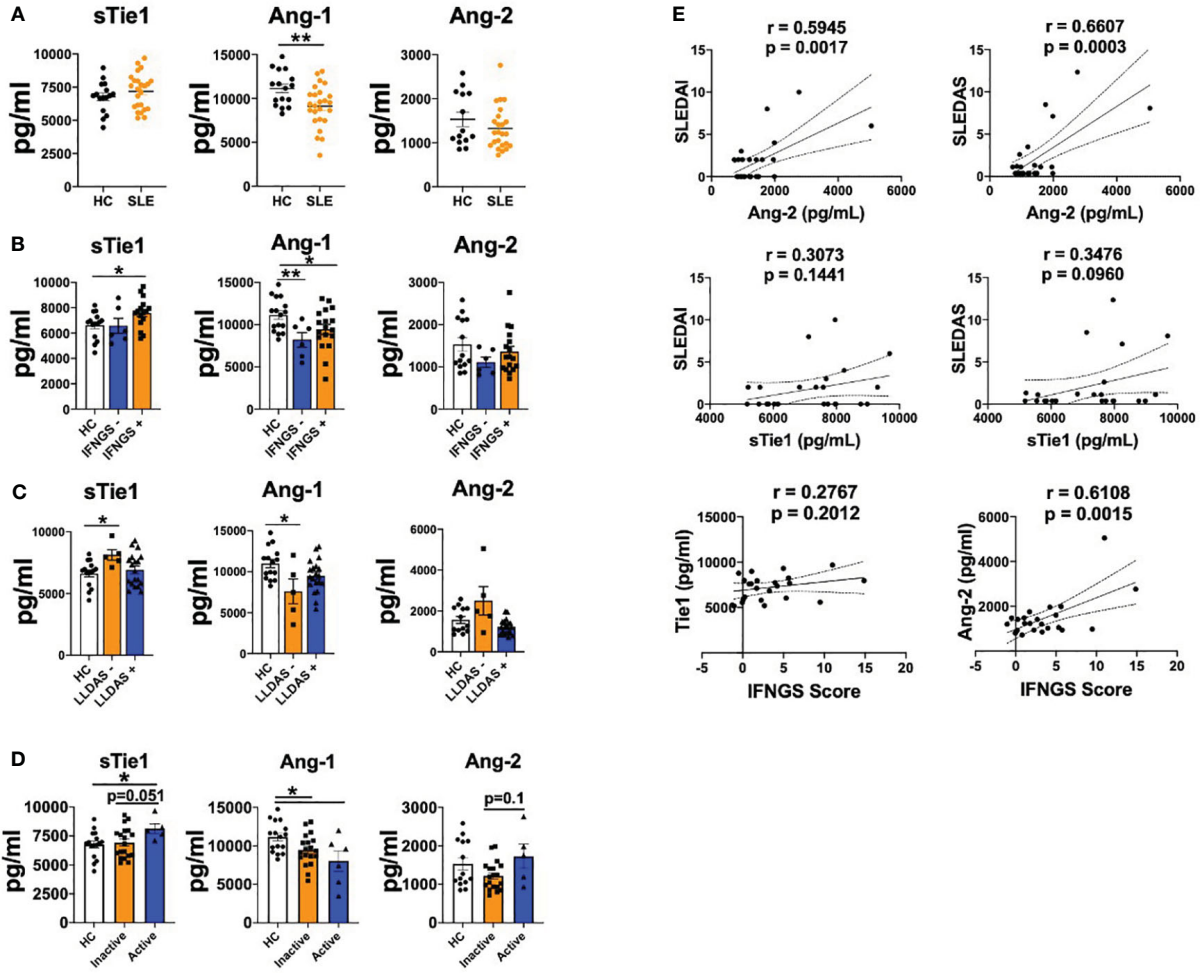
Tie2 signaling is destabilized in patients with SLE. A-D. sTie1, Ang-1 and Ang-2 levels (pg/mL) in healthy controls (HC) and patients with SLE **(A)**; stratified by IFN signature activation **(B)**; or low disease activity [LLDAS] **(C)**; or disease activity regarding SLEDAS score [Active/Inactive] **(D, E)** Correlations between sTie1, Ang-2 levels and SLEDAI, SLEDAS, IFNGS scores. Bars show the mean ± SEM. * = p< 0.05; ** = p< 0.01. SLEDAI: Systemic Lupus Erythematosus Disease Activity Index; SLEDAS: Systemic Lupus Erythematosus Disease Activity Score; LLDAS: Lupus Low Disease Activity Score; Ab: patients positive for at least one autoantibody (anti-cardiolipin, anti-β2-glicoprotein GP 1, anti-dsDNA). Unpaired T test, r Pearson correlation and Kruskal-Wallis statistical tests were used.

We first determined the serum levels of Ang-1, Ang-2 and sTie1 in our cohort of patients with SLE. In line with previous studies (17), Ang-1 levels were significantly (p=0.0069) decreased in patients with SLE compared to healthy controls (HC), but Ang-2 levels were not elevated in our cohort (**Figure 1A**). In addition, sTie1 levels showed a trend towards increased levels in patients with SLE. Since type I IFN play a major role in the pathogenesis of SLE, we analysed the expression of IFN signature genes to characterise our patient cohort and the association of Ang-1, Ang-2 and sTie1 levels according to this IFN signature. Compared to HC, Ang-1 levels were reduced in both IFN signature negative and positive patients, while sTie1 levels were significantly (p=0.0265) elevated in IFN signature-positive patients (**Figure 1B**). In the case of Ang-2, there were no differences according the IFN signature; however, Ang-2 levels positively correlated with this signature (**Figure 1E**).

We also determined the possible associations between the expression of these mediators and the clinical characteristics of patients. Patients positive for at least one autoantibody (anti-cardiolipin, anti-β2-glicoprotein GP1, anti-dsDNA) presented a trend towards increased sTie1 and Ang-2 levels compared to HC or autoantibody-negative patients. Also, patients with low complement system levels showed a trend of increased levels of sTie1 and Ang-2 compared to HC and patients with higher complement levels (**Supplementary Figure S2**). When we analysed SLE activity states (LLDAS and DORIS-21), we observed that patients who did not fulfil the LLDAS definition showed a trend towards higher levels of Ang-2 and significantly (p=0.0354) increased sTie1 levels compared to HC and patients that fulfilled these criteria, while Ang-1 levels were significantly (p=0.0445) reduced (**Figure 1C**). Also, similar results were found in patients that did not fulfil DORIS-21 (**Supplementary Figure S2**). Patients with active SLE (SLEDAI > 7.64) presented higher levels of sTie1 and Ang-2 and decreased levels of Ang-1 (**Figure 1D**). Importantly, SLEDAI and SLEDAS scores positively correlated with Ang-2 and sTie1, but differences were only significant (p=0.027 and p=0.0003) for Ang-2 (**Figure 1E**).

In conclusion, these data confirm that Tie2 signalling mediators are dysregulated in patients with SLE and are associated with the IFN signature and clinical characteristics.

### 3.2 Type I IFN promote Ang-2 production by SLE monocytes and deregulates Ang-1/Ang-2 release in different human and mice kidney datasets

**Figure 2:**
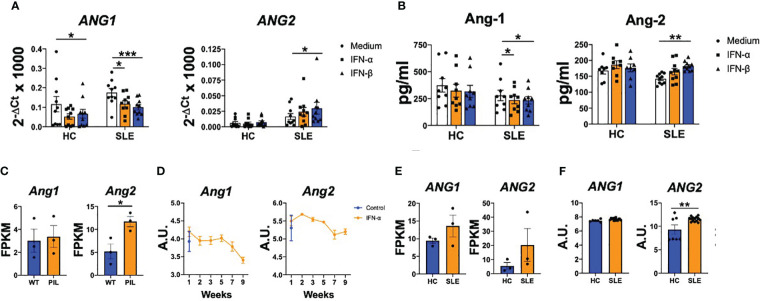
Type I IFN modulate angiopoietins production in monocytes from patients with SLE monocytes and in kidneys of patients with lupus nephritis. **(A, B)** Ang-1 and Ang-2 mRNA **(A)** and protein **(B)** levels by monocytes of healthy controls (HC) and SLE patients stimulated with IFN-α and IFN-β (1000 IU/mL) for 24 (h) Data are shown as relative quantity compared to Jnon-stimulated monocytes. **(C, D)** Ang1 and Ang2 mRNA expression in kidney from wild type (WT) and pristane-induced SLE mice model [PIL, GSE173767 **(C)**] and from lupus nephritis mice model untreated or treated with IFN-α [GSE86423 **(D)**]. **(E, F)** ANG1 and ANG2 mRNA expression in kidney from HC and SLE patients -GSE157293- **(E)** or from kidneys of HC and lupus nephritis patients -GSE112943- **(F)**. Data are shown as Fragments Per Kilobase per Million Mapped Fragments (FPKM) or arbitrary units (A.U.). Bars show the mean ± SEM. * = p<0.05; ** = p<0.01; *** = p<0.001 . Two-way ANOVA and unpaired T statistical tests were used.

We next sought immune cell types responsible of the dysregulated angiopoietins levels observed in patients with SLE. As previous studies from our group showed that IFN- α stimulation induced Ang-2 production by monocytes from systemic sclerosis patients (31), we determined the effect of IFN- α and IFN-β on SLE monocytes. Type I IFN stimulation significantly (p values ranged between 0.0009 and 0.0383) down-regulated *ANG1* expression in both HC and SLE monocytes, while *ANG2* expression was significantly (p=0.0463) up-regulated only in monocytes from patients (**Figure 2A**). We confirmed these findings at the protein level, as Ang-1 levels significantly (p=0.0468 and p=0.0468) decreased and Ang-2 levels significantly (p=0.0017) increased under type I IFN stimulation in SLE monocytes (**Figure 2B**).

Also, it was analysed whether angiopoietins are impaired in affected tissues, using publicly available databases. *Ang2* expression was modulated on kidney samples from the pristane-induced murine model (GSE173767, **Figure 2C**). Importantly, in a murine model of lupus nephritis (NZB X NZW F1 (BWF1)), the treatment with IFN-α reduced the *Ang1* expression along the time course of the disease, while the *Ang2* levels were up-regulated at the first week of the model (**Figure 2D**). We also determined the Ang-1 and Ang-2 expression in kidneys of lupus nephritis patients (GSE157293 and GSE112943). In these patients, *ANG1* expression was similar compared to HC, while *ANG2* expression was significantly (p=0.0052) up-regulated (**Figures 2E, F**). Altogether, these data show that type I IFN modulate the expression of Ang-1 and Ang-2 in circulating cells and both mediators are also dysregulated at the affected tissues.

### 3.3 Type I IFN destabilizes Tie2 signalling in endothelial cells

**Figure 3:**
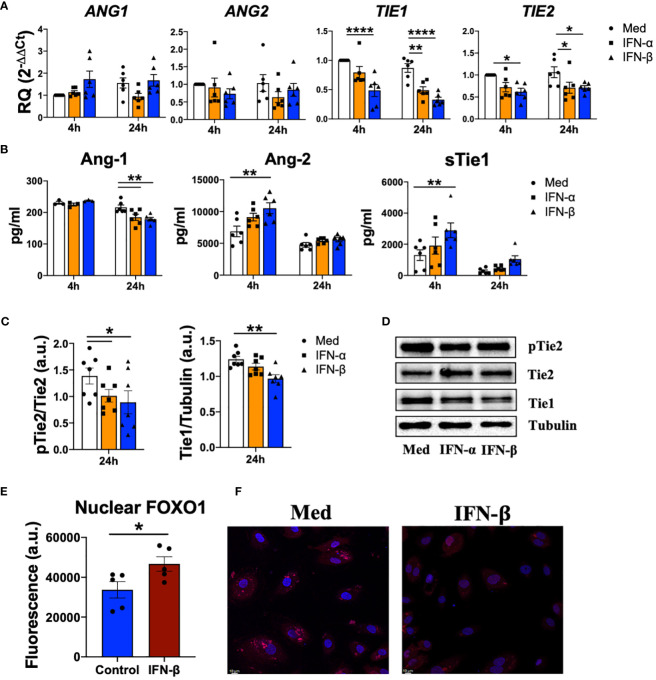
Type I IFN destabilize Tie2 signaling in endothelial cells. **(A, B)** ANG1, ANG2, TIE1, TIE2 mRNA expression and Ang-1, Ang2, sTie1 protein levels in human umbilical vein endothelial cells (HUVEC) stimulated with IFN-α and IFN-β (1000 IU/mL) for 4 and 24 h. Data are shown as relative quantity (RQ) compared to non-stimulated HUVEC of Sc HUVEC. **(C, D)** Densitometric analysis **(C)** and representative immunoblot **(D)** of Tie1 expression and Tie2 activation in HUVEC stimulated with IFN-α and IFN-β (1000 IU/mL) for 24 h. Data is shown as arbitrary units (a.u.) with respect to tubulin expression. **(E, F)** Fluorescence analysis **(E)** and representative picture of FOXO1 nuclear translocation in HUVEC **(F)** stimulated with IFN-β (1000 IU/mL) for 15 min. Data is shown as fluorescence arbitrary units (a.u.). Bars show the mean ± SEM. * = p<0.05; ** = p<0.01; **** = p<0.0001. Two-way ANOVA, Kruskal-Wallis and Unpaired T statistical tests were used.

Next, we determined whether type I IFN modulate Tie2 signalling in endothelial cells. IFN-α and IFN-β did not regulate the expression of *ANG1* or *ANG2*, but significantly (p values ranged between 0.0207 and<0.0001) down-regulated *TIE1* and *TIE2* expression (**Figure 3A**). However, type I IFN significantly (p=0.0062 and p=0.0015) reduced the secretion of Ang-1, while significantly (p=0.0042) enhanced Ang-2 secretion and the release (p=0.0025) of sTie1 (**Figure 3B**). Furthermore, we confirmed this release, since Tie1 protein levels were significantly (p=0.0060) decreased upon IFN-β stimulation, indicating the cleavage of this protein (**Figure 3C**).

To determine whether type I IFN also modulate Tie2 signalling, we analysed the phosphorylation of Tie2 and we observed a significant (p=0.0462 and p=0.0178) reduction after IFN-α and IFN-β stimulation (**Figures 3C, D**). Interestingly, IFN-β modulates FOXO1 translocation, a transcription factor involved in Tie2 signalling (15) by significantly (p=0.0454) increasing FOXO1 nuclear levels (**Figures 3E, F**).

Overall, these results show how type I IFN destabilize Tie2 signalling in endothelial cells.

### 3.4 IFN-β disrupts normal function in angiogenesis and viability through Tie1 and IFNAR1 receptors

**Figure 4:**
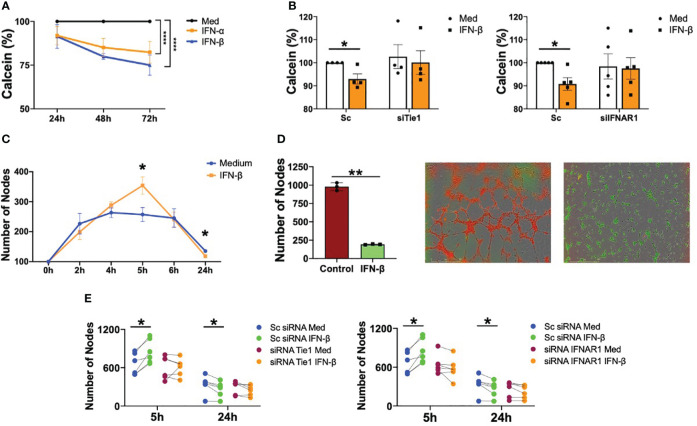
Type I IFN impair viability and angiogenesis in a IFNAR1 and Tie2 signaling dependent manner. **(A)** Viability of human umbilical vein endothelial cells (HUVEC) stimulated with IFN-α and IFN-β (1000 IU/mL) for 24, 48 and 72 (h) Data are shown as percentage with respect to non-stimulated cells. **(B)** Viability of HUVEC transfected with scramble (Sc), Tie1 or IFNRA1 siRNA and stimulated with IFN-β (1000 IU/mL) for 48 (h) Data are shown as percentage with respect to non-stimulated Sc siRNA-transfected cells. **(C, D)** Tube formation in HUVEC stimulated with IFN-β (1000 IU/mL) at the indicated time points **(C)** or tube formation at 5h in HUVEC control (red) primed with IFN-β IFN-β (1000 IU/mL) for 24 h (green) **(D)**. Data are shown as number of nodes. **(E)** Tube formation in HUVEC transfected with scramble (Sc), Tie1 or IFNRA1 siRNA and stimulated with IFN-β (1000 IU/mL) for 5 and 24 (h) Data are shown as number of nodes. Bars show the mean ± SEM. * = p<0.05; ** = p<0.01; **** = p<0.0001. Two-way ANOVA and Paired T statistical tests were used.

We next determined the functional consequences of type I IFN on endothelial cells dysfunction and the implication of Tie2 signalling. We showed that both IFN-α and IFN-β are able to significantly (p<0.0001) decrease the viability of HUVEC, confirming the role of these cytokines on endothelial integrity (**Figure 4A**). Given that we have demonstrated the role of type I IFN destabilizing Tie2 signalling through the cleavage of Tie1, we next knocked down the expression of Tie1 and IFNAR1 receptors in HUVEC and we determined the functional consequences. Results showed that both Tie1 and IFNAR1 silencing expression abrogated the reduction in viability mediated by IFN-β (**Figure 4B**).

It was also demonstrated that IFN-β regulates EC tube formation. Angiogenesis was significantly (p=0.0151) increased at 5 hours of stimulation, but significantly (p=0.0401) decreased at 24 h (**Figure 4C** and **Supplementary Figure S3**). Further interrogation was performed with a novel experimental approach to understand the effect of chronic exposure to this cytokine, because a hallmark of patients with SLE is the sustained maintenance of the inflammatory environment (32). IFN-β pre-treatment for 24 h significantly (p=0.0012) inhibited the ability of HUVEC to form tubes compared to non-pre-treated cells (**Figure 4D**). Next, we determined the role of IFNAR1 and Tie2 signalling on IFN-β-mediated angiogenesis. As expected, IFN-β significantly (p=0.0126) promoted tube formation at 5 h and significantly (p=0.048) inhibited this process after 24 hours in Sc condition. However, this effect was reversed with both Tie1 and IFNAR1 silencing, demonstrating that these receptors are involved in IFN-β-mediated angiogenesis (**Figure 4E**).

In short, we demonstrated that IFN-β has a dual role in the regulation of angiogenesis in endothelial cells, inducing tube formation at short times but inhibiting angiogenesis at long exposure times. Importantly, this role was mediated by Tie2 signalling destabilization.

### 3.5 IFN-β modulates cell adhesion and metabolism related genes

**Figure 5:**
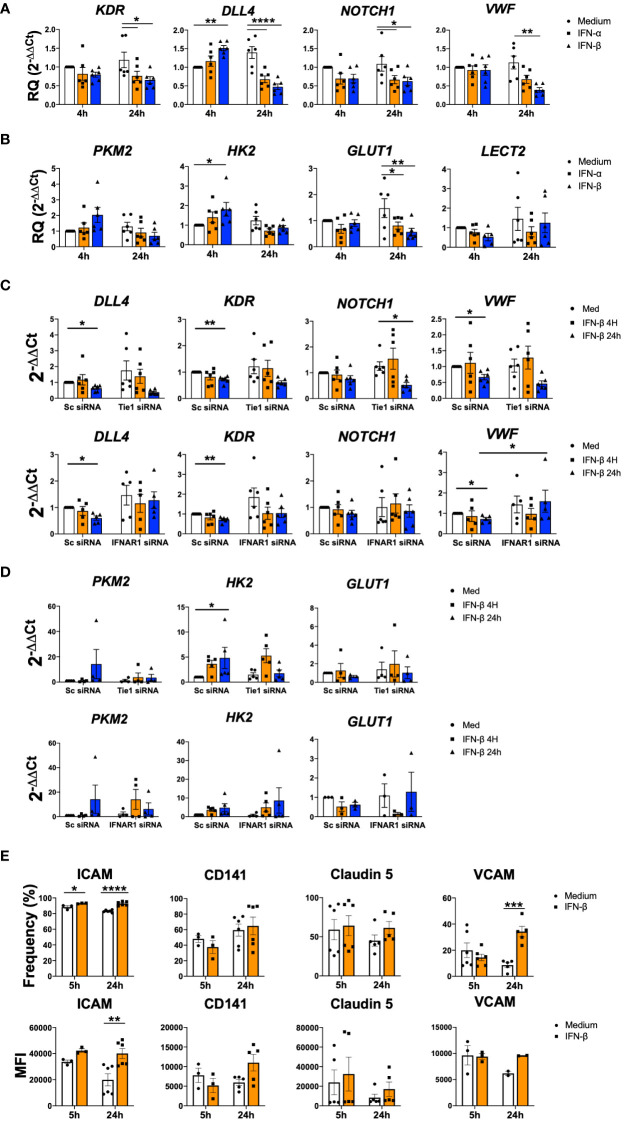
IFN-β modulates cell adhesion and metabolism related genes. **(A-D)**. *KDR, DLL4, NOTCH1, VWF, PKM2, HK2, GLUT1* and *LECT2* expression in non-transfected **(A, B)** or HUVEC transfected with scramble (Sc), Tie1 or IFNRA1 siRNA and stimulated with IFN-α or IFN-β (1000 IU/mL) for 4 and 24 h **(C, D)**. Data are shown as relative quantity compared to non-stimulated Sc HUVEC. **(E)** ICAM, CD141, Claudin5 and VCAM adhesion protein levels in Matrigel embedded HUVEC and stimulated with IFN-β (1000 IU/mL) for 5 and 24 h. Data are shown as percentage of cell and Median Fluorescence Intensity (MFI). Bars show the mean ± SEM. * = p< 0.05; ** = p< 0.01; *** = p< 0.001; **** = p< 0.0001. Two-way ANOVA statistical test was used.

Next, we analysed the regulation of genes involved in angiogenesis and cell adhesion. At 4 h, type I IFN stimulation significantly (p=0.0027) induced the expression of *DLL4*; however, both IFN-α and IFN-β significantly (p values ranged between<0.0001 and 0.0481) down-regulated the expression of *KDR*, *DLL4*, *NOTCH1*, *VWF* and *LECT2* at 24 h (**Figures 5A, B**). Remarkably, expression of metabolic genes such as *PKM2, HK2* and *GLUT1* also showed a trend to increase at early time points and to decrease at late time points (**Figure 5B**).

We further explored the role of Tie1 and IFNAR1 receptors. Expression levels in scramble conditions decreased significantly (p values ranged between 0.0066 and 0.0318) at 24 h for *DLL4*, *KDR*, *VWF* and *NOTCH1*. Tie1 silencing did not modulate the down-regulation of these genes, while IFNAR1 silencing abrogated it, showing the involvement of this receptor in the regulation of these mediators (**Figure 5C**).

In the case of metabolic genes, IFN signalling was not involved in their expression; however, Tie1 silencing partially abolished the IFN-β up-regulated expression of *PKM2* and *HK2* (**Figure 5D**).

We also analysed the expression of several adhesion proteins during angiogenic process (33), where we found a significant (p<0.0001 and p=0.0007) increase in ICAM and VCAM after 24 h of stimulation, as well as an increasing trend in CD141 and Claudin5 (**Figure 5E**).

Overall, this data suggests that IFN signalling also modulate other pathways involved in angiogenesis as well as cell metabolic and cell adhesion.

### 3.6 SLE serum induces endothelial cell destabilization in a Tie2-dependent manner

**Figure 6:**
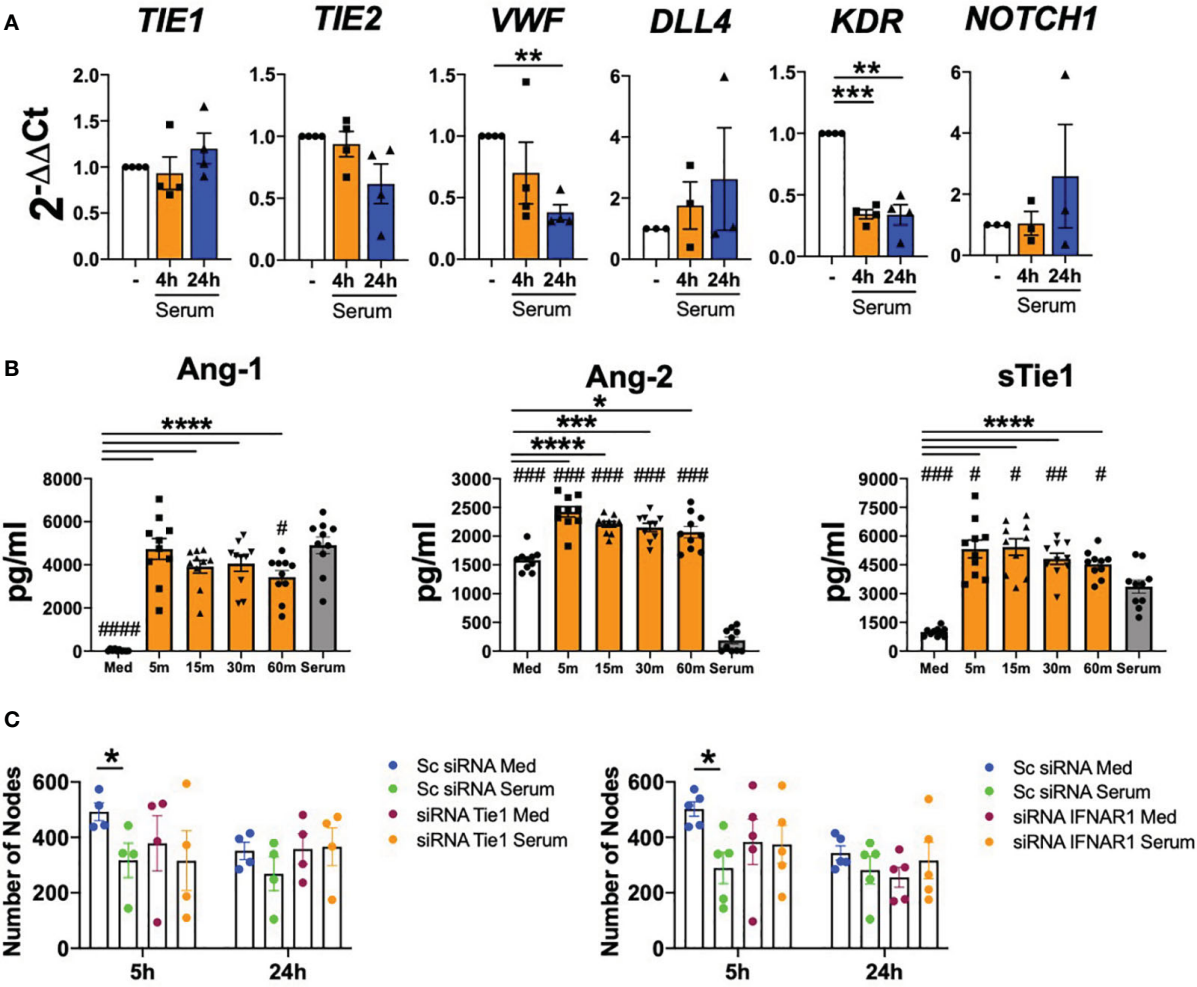
SLE serum induces endothelial cell destabilization in a Tie2-dependent manner. **(A)** *TIE1, TIE2, VWF, DLL4, KDR* and *NOTCH1* mRNA expression in HUVEC under SLE serum stimulation at 20% (v/v) for 4 and 24 (h) **(B)**. Ang-1, Ang-2, sTie1 protein levels in HUVEC stimulated with the serum of SLE patients 20% (v/v) at the indicated time points. **(C)** Tube formation in HUVEC transfected with scramble (Sc), Tie1 or IFNRA1 siRNA and stimulated with SLE serum patients (20% v/v) for 5 and 24 (h) Data are shown as number of nodes. Bars show the mean ± SEM. * = p< 0.05; ** = p< 0.01; *** = p< 0.001 and **** = p< 0.0001. # = p< 0.05; ## = p< 0.01; ### = p< 0.001 and #### = p< 0.0001 compared to serum. One-way and Two-way ANOVA statistical tests were used.

Finally, we determined whether type I IFN present in the serum of patients with SLE are able to induce Tie2 destabilization and endothelial cells dysfunction. SLE serum down-regulated the expression of *TIE2*, *VWF* (p=0.0038) and *KDR* (p=0.0006 and p=0.0067) (**Figure 6A**).

Importantly, serum stimulation significantly (p values ranged between 0.0148 and<0.001) induced Ang-2 and sTie1 secretion at early time points and this induction was not due to Ang-2 and sTie1 levels present in the serum of patients. However, Ang-1 induction was not observed, as we did not find differences between serum-stimulated cells and the Ang-1 levels present in the serum (**Figure 6B**).

Furthermore, we studied serum effect in angiogenesis with HUVEC in which Tie1 and IFNAR1 receptors were silenced. Results obtained in the scramble conditions showed that SLE serum significantly (p=0.0455) reduced the angiogenic capacity of endothelial cells at 5 h and non-significantly at 24 h. Remarkably, this effect of serum was reversed after Tie1 and IFNAR1 silencing (**Figure 6C** and **Supplementary Figure S4**), demonstrating that the effect of serum in angiogenesis depends on Tie2 and type I IFN signalling pathways.

## 4 Discussion

In this study, we demonstrate that, in SLE pathogenesis, type I IFN contribute to the endothelial destabilization in a Tie2-dependent manner. Kumpers et al. (17) showed in patients with SLE that Ang-1 levels were decreased compared to controls and Ang-2 levels increased, showing the imbalance of Tie2 signalling in SLE. Our results show the same imbalance, finding a significant reduction in Ang-1 levels and, for the first time as far as we know, elevated sTie1 serum levels in patients with SLE. Interferon gene signature (IFNGS) is used to detect type I IFN associated status and has been reported as a biomarker of activity in SLE, since it is increased with respect to healthy controls (34, 35). Our cohort showed a positivity of 75% (**Supplementary Figure S5A**), which is in the range reported among patients with SLE (52-87%) (34) and positively correlated with the clinical activity scores SLEDAI and SLEDAS (**Supplementary Figure S5B**). Interestingly, sTie1 levels were significantly higher in IFNGS positive group and Ang-1 levels were significantly lower than in controls for both IFNGS groups, confirming the role of type I IFN on Tie2 dysregulation. Stratification of patients based on disease activity, autoantibodies presence and activation of complement system showed interesting associations with Tie2 imbalance. Remarkably, patients with hypocomplementemia, which is due to having undergone recent complement activation and subsequent drop in levels, showed higher sTie1 and Ang-2 values. This complement activation is associated with an increased risk of thrombosis and cardiovascular events (36, 37) and we show here the association between complement activation and Tie2 destabilization. Here, we also found that clinical values were associated and correlated with Tie2 destabilization, being the studied proteins potential biomarkers of SLE prognosis and activity. Moreover, in line with previous results reported, Ang-2 levels were correlated with clinical disease activity. This likely explains the lack of differences in the Ang-2 levels between HC and our cohort of patients, as most of them had low disease activity. In fact, Ang-2 levels were elevated in patients with higher activity (17).

Altogether, these data suggest that Ang-1, Ang-2 and sTie1 may be potential biomarkers of low disease activity and remission states, although further studies in bigger cohorts are needed for validating these findings.

Based in previous works from patients with systemic sclerosis, we also have studied the role of monocytes as angiopoietin-expressing cells (31). Type I IFN down-regulated the expression of Ang-1 in SLE monocytes, while enhanced Ang-2 levels. This modulation has functional consequences, as the stimulation of HUVEC with the supernatant of SLE monocytes stimulated with IFN-β (conditioned media -CM_IFN-β_-) reduced the activation of Tie2 and induced a defective angiogenesis, compared to non-stimulated cells, or stimulated with the conditioned media of unstimulated monocytes (**Supplementary** **Figure 6**). Since it was recently reported that the main producers of type I IFN are not pDCs, but other cell populations on macrophages such as neutrophils and macrophages (38), we also analysed the effect of both Ang-1 and Ang-2 on type I IFN genes expression and we found that Ang-1 decreases the expression of *IFNA* and *IFNB* (**Supplementary Figure S7**). Altogether, these results show an interplay between type I IFN and angiopoietins in myeloid cells from patients with SLE and demonstrate a functional role on endothelial stability.

TNF, VEGF and LPS were previously identified as mediators capable of initiating Tie2 destabilisation (16, 39). Numerous studies have shown the involvement of type I IFN in vascular destabilization associated with pathologies such as thrombosis, viral infections and autoimmune diseases (12, 40, 41), but whether type I IFN induce endothelial dysfunction through Tie2 signalling had not yet been elucidated. Here we show that type I IFN destabilize Tie2 signalling by inducing Tie1 cleavage, reducing of Tie2 activation and translocating FOXO1 to the nucleus, leading to the secretion of Ang-2 and the reduction of Ang-1 expression. The functional consequences were the reduction of endothelial cell viability and the induction of defective angiogenesis. Even though angiogenesis promotion was previously described by IFN-α (42), here we showed for the first time, to our knowledge, that type I IFN have a dual role time-dependent in angiogenesis, as IFN-β induced angiogenesis at early times and inhibited tube formation at late times. These data indicate that the induction of angiogenesis by IFN-β is defective and unstable, suggesting that a chronic exposure to this cytokine, like happens in SLE, promotes pathological angiogenesis, triggering endothelial cell damage and dysfunction, which contribute to the apparition of CVE in patients with SLE. Importantly, both the reduced viability and the defective angiogenesis were abrogated after the silencing of Tie1 expression, pointing out the important role of Tie2 signalling on the type I IFN-mediated EC dysfunction.

Interestingly, IFN-α and IFN-β also regulate endothelial metabolism. Previous works have shown that this metabolism can be modified by inflammatory mediators (43). For instance, TNF induces mitochondrial respiration and LPS enhances glycolysis in endothelial cells (44, 45). Our 2P-FLIM results showed an increase at short times of oxidative phosphorylation (OxPhos) metabolic activity in junction cells in comparison to tube cells when stimulated with IFN-β at 5 h, being the opposite in control cells (**Supplementary Figure S8**). These results support our finding of pathological angiogenesis, since it is expected that, when treated with IFN-β, junction cells are more metabolically active, given that this is where new tube formation is initiated.Of special interest, we showed that SLE serum is also able to reduce the angiogenic capacity of endothelial cells. Outstandingly, it was determined that this modulation is in turn mediated by Tie2 and type I IFN signalling pathways, suggesting a key contribution of type I IFN present in the serum of patients with SLE (46, 47) on the cardiovascular events observed in these patients.

It is important to note that Tie2 dysregulation was also observed in kidneys of lupus nephritis (LN) patients. As type I IFN are also increased in kidneys of these patients and microvascular lesions are frequent in LN, it is tempting to speculate that these lesions are due to the type I IFN-mediated Tie2 destabilization (48).

In this study we also found that type I IFN regulate other angiogenic-related genes, such as *NOTCH1* and *DLL4*. Notch-1 is a transmembrane receptor with several ligands such as Delta-like ligands 1-4 (Dll1-4) and Jagged 1-2. It was reported that Tie2 signalling promotes Notch-1 pathway activation, which is key for the normal function of endothelium and protecting ECs from apoptosis (49). Specifically, Dll4 activates Notch-1 signalling to guarantee homeostatic angiogenesis and vascular stability (50). In this study, we demonstrate that type I IFN reduce *DLL4* and *NOTCH1* expression levels, but this effect is not dependent on Tie2 signalling, suggesting that type I IFN may induce vascular dysfunction also through other signalling pathways. However, our data suggest that Tie2 signalling might modulate the expression of metabolism-related genes, although further experiments are needed for confirming this mechanism.

Finally, we found a modulation towards an increase on adhesion molecules in EC during tube formation. To our knowledge, this is the first time that flow cytometry is performed in HUVEC while cells are in the angiogenic process, allowing a more precise understanding of the EC phenotype in tube formation.

A potential limitation to consider in this work is the lack of direct association between endothelial damage and CVE. However, different studies have widely reported that endothelial damage triggers the axis endothelial dysfunction – inflammation – cardiovascular failure (51, 52). Angiopoietins are involved in this axis, as increased levels of Ang-1 have been described as protective and Ang-2 levels as inducers of cardiovascular events (19, 53, 54). Thus, we demonstrated that type I IFN induce an imbalance in angiopoietins levels, showing a direct effect of interferons in cardiovascular events.

Of special interest, anifrolumab is a drug that targets IFNAR recently approved for the treatment of SLE (55, 56). Recent studies have shown that anifrolumab reduces cardiometabolic disease markers, cytokines associated with vascular dysfunction and, importantly, Ang-2 (57, 58). Therefore, anifrolumab may have a cardiovascular protective role due to the stabilization of the Tie2 signalling, but further studies are needed.

In conclusion, our findings postulate Tie1 and Tie2 ligands as potential biomarkers of states of low disease activity and remission in patients with SLE and identified a new molecular mechanism of endothelial destabilization mediated by type I IFN and Tie2 signalling pathways, which may contribute to cardiovascular events in patients with SLE.

## Data availability statement

The datasets presented in this study can be found in online repositories. The names of the repository/repositories and accession number(s) can be found below: https://www.ncbi.nlm.nih.gov/geo; GSE173767, GSE86423, GSE157293, GSE112943.

## Ethics statement

The studies involving humans were approved by Ethics Committee of Galicia (study number 2020/158). The studies were conducted in accordance with the local legislation and institutional requirements. The participants provided their written informed consent to participate in this study.

## Author contributions

CR-V: Conceptualization, Data curation, Formal analysis, Methodology, Validation, Writing – original draft, Writing – review & editing, Investigation. SM-R: Data curation, Formal analysis, Methodology, Writing – review & editing. BM-F: Data curation, Formal analysis, Writing – review & editing. IA-G: Data curation, Methodology, Writing – review & editing. CM: Data curation, Methodology, Writing – review & editing. DV: Conceptualization, Writing – review & editing. AF: Data curation, Formal analysis, Methodology, Writing – review & editing. UF: Conceptualization, Supervision, Writing – review & editing. JR: Conceptualization, Formal analysis, Investigation, Writing – review & editing. SG: Conceptualization, Data curation, Formal analysis, Funding acquisition, Methodology, Project administration, Resources, Supervision, Validation, Writing – original draft, Writing – review & editing.
