# Supplementary material for: Type I Interferons induce endothelial destabilization in Systemic Lupus Erythematosus in a Tie2-dependent manner
Source: Front Immunol. 2023 Dec 14;14:1277267. doi: 10.3389/fimmu.2023.1277267 (PMC10756137; doi:10.3389/fimmu.2023.1277267)
Supplement: Supplementary file 1 [file DataSheet_1.docx]

**Supplementary Figure 1.**

**Supplementary Figure S1. Efficiency of cell transfection in HUVEC.** **A-B.** mRNA, protein (A) and representative blots (B) of Tie1 and IFNAR1 expression in HUVEC transfected with scrambled control (Sc), Tie1 or IFNAR1 siRNA. Data is shown as expression respect to Sc siRNA-transfected cells or arbitrary units (a.u.) with respect to tubulin expression. Bars show the mean ± SEM. * = p < 0.05, ** = p < 0.01, *** = p < 0.001. Paired T statistical test was used.

**Supplementary Figure 2.**

**Supplementary Figure S2. SLE clinical characteristics are associated with Tie2 disbalance**. sTie1, Ang-1 and Ang-2 levels (pg/mL) in patients with SLE stratified by presence of autoantibodies [Ab] or anti-cardiolipin [𝛼-ACA] or anti-dsDNA [𝛼-dsDNA] autoantibodies or complement system activation [Comp] or low activity of disease [DORIS-21]. Bars show the mean ± SEM. * = p< 0.05. anti-ds-DNA: anti double stranded DNA; DORIS-21: Definitions of Remission In SLE. Kruskal-Wallis statistical test was used.

**Supplementary Figure 3.**

**Supplementary Figure S3. IFN-β effect in angiogenesis**. Representative images of tube formation assay in control (Med) and IFN-β (1000 IU/mL) stimulated HUVEC at the indicated time points.

**Supplementary Figure 4.**

**Supplementary Figure S4**. **SLE Serum effect in angiogenesis.** Representative images of tube formation in HUVEC transfected with scramble (Sc), Tie1 or IFNRA1 siRNA and stimulated with SLE serum patients (20% v/v) at the indicated time points.

**Supplementary Figure 5.**

**Supplementary Figure S5. IFN signature expression in HC and SLE cohorts and its correlation with SLEDAI and SLEDAS**. **A.** Z score values of IFN signature in peripheral blood mononuclear cells of healthy controls (HC) and SLE patients. **B.** Correlations between IFNGS score and SLEDAI and SLEDAS scores. Bars show the mean ± SEM. SLEDAI: Systemic Lupus Erythematosus Disease Activity Index; SLEDAS: Systemic Lupus Erythematosus Disease Activity Score. **=p< 0.01. Unpaired T statistical test and r Pearson correlation were used.

**Supplementary Figure 6.**

**Supplementary Figure S6. Monocyte SLE supernatants regulate angiogenesis and Tie2 activation**. **A**. Tube formation (A), densitometric analysis (B) and representative immunoblot (C) of Tie2 activation in control HUVEC (Med) and stimulated with supernatants non-treated (Conditioned Media, CM_med_) or IFN-β treated (CM_IFN-β_) from SLE monocytes for 5 and 24 h. **B** in HUVEC stimulated with supernatants non-treated (CM_med_) or IFN-β treated (CM_IFN-β_) from SLE monocytes. Data is shown as arbitrary units (a.u.) with respect to tubulin expression. *=p< 0.05. Kruskal-Wallis and Mann-Whitney statistical tests were used.

**Supplementary Figure 7.**

**Supplementary Figure S7. Ang-1 modulates *IFNA* and *IFNB* expression in SLE macrophages**. *IFNA* and *IFNB* mRNA expression in macrophages from SLE patients stimulated with Ang-1 (200 ng/ml) or Ang-2 (200 ng/ml) for 4 and 24 h. Data are shown as relative quantity compared to non-stimulated macrophages. Bars show the mean ± SEM.

**Supplementary Figure 8.**

**Supplementary Figure S8. IFN-β effect in HUVEC angiogenesis metabolism**. Average lifetime (𝜏_avg_) ﻿of NAD(P)H in angiogenesis (tubes and junctions) in control cells (basal) and under stimulation with IFN-β for 5 h. Ratio basal/IFN-β is represented. Bars show the mean ± SEM. * = p< 0.05. Unpaired T statistical test was used.

**Supplementary Table 1.**

| **Clinical characteristics** | **SLE** | **HC** |
| --- | --- | --- |
| Number Patients | 25 | 16 |
| Female/Male | 23/2 | 14/2 |
| Age, years | 52 (45-62) | 38 (30.5-43) |
| SLEDAI | 1.8 (0-2) |  |
| SLEDAS | 2.15 (0.37-1.32) |  |
| LLDAS | 20 (80%) |  |
| DORIS-21 | 18 (72%) |  |
| **Cardiovascular risk factors** |  |  |
| Hypertension | 5 (20%) |  |
| Smoking | 5 (25%) |  |
| Type 2 Diabetes | 2 (8%) |  |
| Total cholesterol > 200 mg/dL | 8 (32%) |  |
| **Autoantibodies** |  |  |
| Autoantibodies  (positive at least one) | 11 (45.8%) |  |
| anti-dsDNA | 5 (20.8%) |  |
| anti-cardiolipin | 3 (12.5%) |  |
| anti-β2-glicoprotein GP 1 | 4 (16.6%) |  |
| Lupus anticoagulant | 4 (16.6%) |  |
| **Treatments** |  |  |
| No treatment | 2 (8%) |  |
| Glucocorticoids | 12 (48%) |  |
| Hydroxycloroquine | 16 (64%) |  |
| Conventional immunosuppresants | 10 (40%) |  |
| Biologics | 4 (16.6%) |  |

**Supplementary Table S1. Clinical characteristics of SLE and healthy controls (HC) cohorts**. Median and interquartile range are shown. Percentage data represents % of patients of total cohort included in that group. SLEDAI: Systemic Lupus Erythematosus Disease Activity Index; SLEDAS: Systemic Lupus Erythematosus Disease Activity Score; LLDAS: Lupus Low Disease Activity Score; DORIS-21: Definitions of Remission In SLE; anti-ds-DNA: anti double stranded DNA.

**Supplementary Table 2.**

| Gene | Sequence | Length |
| --- | --- | --- |
| TIE1 Fw | ACAATGGTGTCTGCCATGAA | 20 |
| TIE1 Rv | TTCACAAGCCTTCTCACACG | 20 |
| TIE2 Fw | CAGATTGCGCTACAGCTAGG | 20 |
| TIE2 Rv | CCGCGTAAGTGAAGTTCTCA | 20 |
| IFNAR1 Fw | GCGCGAACATGTAACTGGTG | 20 |
| IFNAR1 Rv | GGACCAATCTGAGCTTTGCG | 20 |
| ANG1 Fw | GAATTTTTGGGGTGCTTGAA | 20 |
| ANG1 Rv | GAGAGGCCCAGTAGCTT | 17 |
| ANG2 Fw | TCAGTGGCTAATGAAGCTTGAGA | 23 |
| ANG2 Rv | CCGCTGTTTGGTTCAACAGG | 20 |
| B2M Fw | GATGAGTATGCCTGCCGTGT | 20 |
| B2M Rv | TGCGGCATCTTCAAACCTCC | 20 |
| GAPDH Fw | GCCAGCCGAGCCACATC | 17 |
| GAPDH Rv | TGACCAGGCGCCCAATAC | 18 |
| IFI44L Fw | CCACCGTCAGTATTTGGAATGT | 22 |
| IFI44L Rv | ATTTCTGTGCTCTCTGGCTT | 20 |
| ISG15 Fw | GCCTCAGCTCTGACCC | 16 |
| ISG15 Rv | CGAACTCATCTTTGCCAGTACA | 22 |
| IFIT2 Fw | TGCAAGGACTTGGGAAATG | 19 |
| IFIT2 Rv | CCAGAGTGTGGCTGATGCT | 19 |
| IFIT3 Fw | ACTGTTTCAACGGGTGTTGG | 20 |
| IFIT3 Rv | CCTTGTAGCAGCACCCAATC | 20 |
| MX1 Fw | GCATCCCACCCTCTATTACTG | 21 |
| MX1 Rv | GCATCCCACCCTCTATTACTG | 21 |
| VWF Fw | TGTCCAGTGTGCTGATGACC | 20 |
| VWF Rv | GATGCGGAGGTCACCTTTCA | 20 |
| NOTCH1 Fw | GTCAGGGAAATCGTGCCAGCA | 21 |
| NOTCH1 RV | GCAGATGTAGGAGGCCTCGAA | 21 |
| DLL4 Fw | AACGGGGGACAGTGCCTGAA | 20 |
| DLL4 Rv | AGTTCACAGTAGGTGCCCGTG | 21 |
| HK2 Fw | TTCTTGTCTCAGATTGAGAGTGAC | 24 |
| HK2 Rv | TTGCAGGATGGCTCGGACTTG | 21 |
| GLUT1 Fw | GCCGGCGGAATTCAATGCTG | 20 |
| GLUT1 Rv | AGCATCTCAAAGGACTTGCCC | 21 |
| PKM2 Fw | ATTATTTGAGGAACTCCGCCGCCT | 24 |
| PKM2 Rv | ATTCCGGGTCACAGCAATGATGG | 23 |
| KDR Fw | GTCATTTATGTCTATGTTCAAGATTAC | 27 |
| KDR Rv | CACTAACAGAAGCAATAAATGGAG | 24 |
| LECT2 Fw | GCTGGTCTGATTTCTACCGCA | 21 |
| LECT2 Rv | TCCAGCAGAGCACAAGATGTC | 21 |
| IFNB Fw | CAGCAATTTTCAGTGTCAGAAGC | 23 |
| IFNB Rv | TCATCCTGTCCTTGAGGCAGT | 21 |
| IFNA Fw | GTGAGGAAATACTTCCAAAGAATCAC | 26 |
| IFNA Rv | TCTCATGATTTCTGCTCTGACAA | 23 |

**Supplementary Table S2. Primer list.** Fw: forward; Rv: reverse. Length data in base pairs.
